# Deep Learning-Based Heart Sound Analysis for Left Ventricular Diastolic Dysfunction Diagnosis

**DOI:** 10.3390/diagnostics11122349

**Published:** 2021-12-13

**Authors:** Yang Yang, Xing-Ming Guo, Hui Wang, Yi-Neng Zheng

**Affiliations:** 1 Key Laboratory of Biorheology Science and Technology, Ministry of Education, College of Bioengineering, Chongqing University, Chongqing 400044, China; yangyang99@cqu.edu.cn (Y.Y.); huiw@cqu.edu.cn (H.W.); 2 Department of Radiology, The First Affiliated Hospital of Chongqing Medical University, Chongqing 400016, China

**Keywords:** left ventricular diastolic dysfunction, deep convolutional generative adversarial networks, heart sounds, convolutional neural network, diagnosis

## Abstract

The aggravation of left ventricular diastolic dysfunction (LVDD) could lead to ventricular remodeling, wall stiffness, reduced compliance, and progression to heart failure with a preserved ejection fraction. A non-invasive method based on convolutional neural networks (CNN) and heart sounds (HS) is presented for the early diagnosis of LVDD in this paper. A deep convolutional generative adversarial networks (DCGAN) model-based data augmentation (DA) method was proposed to expand a HS database of LVDD for model training. Firstly, the preprocessing of HS signals was performed using the improved wavelet denoising method. Secondly, the logistic regression based hidden semi-Markov model was utilized to segment HS signals, which were subsequently converted into spectrograms for DA using the short-time Fourier transform (STFT). Finally, the proposed method was compared with VGG-16, VGG-19, ResNet-18, ResNet-50, DenseNet-121, and AlexNet in terms of performance for LVDD diagnosis. The result shows that the proposed method has a reasonable performance with an accuracy of 0.987, a sensitivity of 0.986, and a specificity of 0.988, which proves the effectiveness of HS analysis for the early diagnosis of LVDD and demonstrates that the DCGAN-based DA method could effectively augment HS data.

## 1. Introduction

Left ventricular diastolic dysfunction (LVDD) is a clinical syndrome characterized by inadequate active relaxation and decreased cardiac output, resulting in elevated end-diastolic pressure and possibly alterations in cardiac function [1]. At present, the overall incidence of LVDD in the general population is approximately 30% [2,3,4,5] and has a positive correlation with all-cause mortality. When the manifestations of heart failure (HF) such as dyspnea, edema, and fatigue gradually appear in the LVDD population, it will move towards an irreversible stage and cause progression to HFpEF [6,7], and around half of all HF hospital admissions are accounted for by patients with HFpEF [8]. Therefore, early diagnosis of LVDD is of great significance for preventing the deterioration of cardiac function as well as timely treatment.

LVDD is the result of impaired diastolic function, which expands inadequately the heart lumen and causes insufficient blood to return to the heart. Invasive measurements, such as stiffness index, could be used to evaluate diastolic function, but they are harmful to the human body. At present, echocardiography is the most commonly used non-invasive examination in clinical practice [9], but it cannot effectively detect the early LVDD without organic diseases. Cardiac auscultation is another non-invasive method used in clinical detection of cardiovascular disease, while the application of heart sounds (HS) is limited due to the limited range of human hearing and the high requirement on physicians’ experience [10]. The electronic transducer can digitally record HS, laying the groundwork for subsequent processing and application of HS signal. Furthermore, HS can directly reflect the property of cardiac mechanical activity and provide useful information for early diagnosis of cardiac abnormalities [11].

At present, there are two types of frequently utilized approaches for evaluating cardiovascular diseases utilizing HS: traditional machine learning and deep learning methods. The former need to extract features as input, such as wavelet features [12], Mel-frequency cepstral coefficients [13,14], energy entropy [15], etc. Saraf et al. [16] offered five physiologically-motivated characteristics extracted from HS signals, which might be used to quantify LVDD using echocardiography-like criteria. These characteristics may better reflect the physical meaning of HS. However, selecting useful features requires manual interaction, and machine learning models were built using a small database. Therefore, the robustness cannot be verified. Deep learning, a machine learning extension, has been popular as artificial intelligence has progressed due to its remarkable ability to extract features automatically. For example, He et al. [17] identified normal and abnormal HS in PhysioNet/CinC Challenge 2016 based on a convolutional neural network (CNN). Also on this database, Noman et al. [18] proposed an one-dimensional (1-D) CNN model to learn deep features directly from original HS signals, indicating that CNN has shown great potential in HS classification and diagnosis.

Although the above studies show that cardiovascular diseases can be early diagnosed using HS, the relevant databases are still scarce [19]. Furthermore, the amount of data has a direct influence on the performance of deep learning models. The over-fitting problem will occur if the amount of data is small, resulting in poor performance. Data augmentation (DA) [20] is an elegant solution to this problem. Due to the rigorous requirements for experimental individuals and surroundings, collecting large-scale and high-quality HS signals of LVDD is difficult in this study. As a result, a DA strategy for LVDD diagnosis is necessary.

The generative adversarial networks (GAN) [21] model was used to generate normal HS [22], but it has problems of mode collapse and training instability [23], which often lead to meaningless outputs [24]. Therefore, some researchers have refined it to create a variety of derivatives that have been shown to be successful in generating medical images [25,26]. In addition, these derivatives also have shown potential in generating time series data, such as artificial audio [27] and electroencephalogram signals [28]. The representation of time series data in the two-dimensional (2D) form may be beneficial to machine learning tasks [29,30]. Therefore, to meet the requirements of deep learning models for the data size, we converted the HS signals into 2D spectrograms by short-time Fourier transform (STFT) [31]. Then, a deep convolutional generative adversarial networks (DCGAN) model, a combination of CNN and GAN, was proposed to automatically generate spectrograms for the HS signals.

This paper’s main contributions are as follows:

- LVDD is a common pathophysiological route leading to various cardiovascular diseases [32], and therefore the analysis of HS to diagnose LVDD is a non-invasive method. In this paper, the real HS signals collected by a digital transducer are binary classified automatically using a 2D CNN model for LVDD diagnosis. In addition, the performance of the CNN model in the three datasets is compared to six deep learning models to find the best model.
- Owing to the restricted availability of relevant labels in prevailing public HS databases, there have been no substantial breakthroughs in categorizing specific forms of heart disease [33]. Therefore, this paper presented a DCGAN-based DA method to expand HS database of LVDD, which could establish an effective dataset for deep learning, drastically minimize the cost of real data collection, and improve LVDD diagnosis performance.

## 2. Materials and Methods

**Figure 1:**
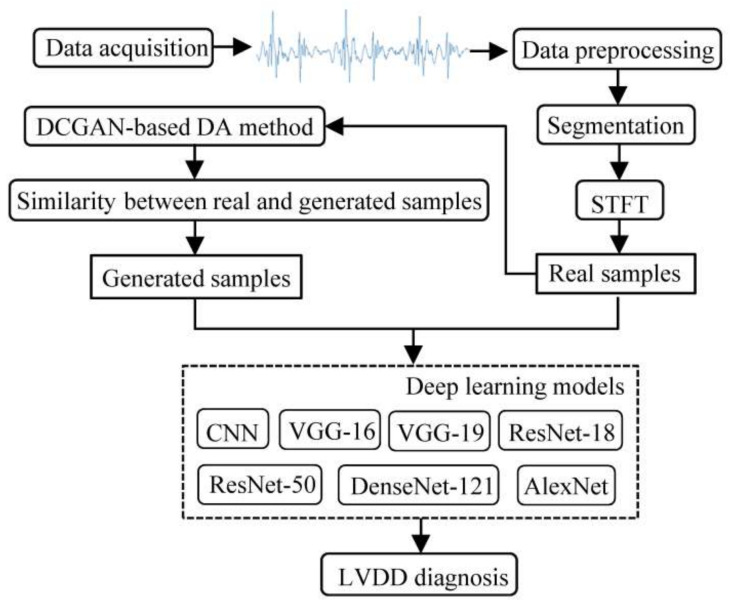
A flow diagram of this paper. The CNN is the proposed model, and the others are the compared models.

The flow diagram of the proposed method is depicted by Figure 1 for automatically diagnosing LVDD, which is explained in the following subsections.

### 2.1. Data Acquisition

All subjects were assessed by cardiologists and completed informed consent forms prior to participation in this study. The day before HS signal collection, the echocardiographic examinations were performed with those patients in the supine position by professional doctors, and the equipment type is ACUSON X300 with the P2-4 phased array ultrasound probe. All subjects had their left ventricular ejection fraction (LVEF) assessed using the biplane Simpson method in the apical four-chamber view. Some Doppler echocardiography indices are shown in Table 1.

A total of 92 HS signals were acquired from the First Affiliated Hospital of Chongqing Medical University by the multi-channel physiological signal acquisition system (RM-6240BD) and HS transducer (XJ102) with the sampling frequency at 8000 Hz, recorded on the apex position of the heart. The HS transducer utilizes piezoelectric-transducer with high-sensitivity, which converts the vibratory HS signal into electrical signal. Then, it is processed by the preamplifier, bandpass filter, 50Hz notch filter, and other circuits of the acquisition system and sent to the single-chip microcomputer for storage after analog-to-digital (A/D) conversion. Finally, the acquisition system is connected to the computer, and the waveform is exhibited on the LCD screen. The collection time was approximately five minutes.

According to the recommendations for the evaluation of left ventricular diastolic function by echocardiography from the American Society of Echocardiography and the European Association of Cardiovascular Imaging (ASE/EACVI) [34], the subjects were divided into: (i) LVDD group, 30 subjects with LVDD, including G1, G2, G3; (ii) control group, 41 subjects with normal diastolic dysfunction, G0. The remaining 21 HS signals could not determine LVDD and were excluded from the study, GIND.

### 2.2. Data Preprocessing

**Figure 2:**
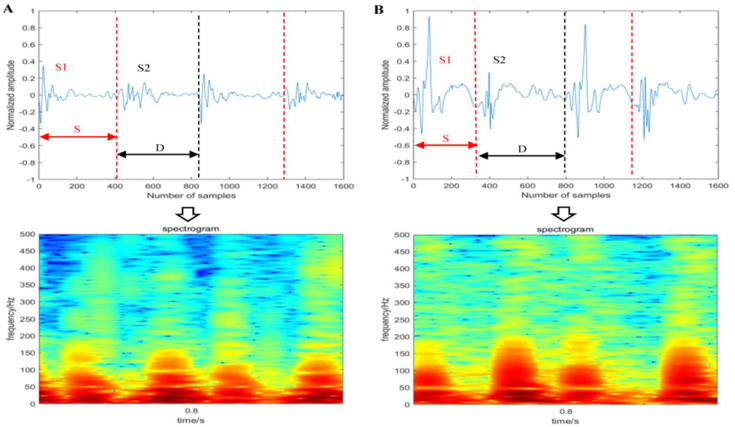
The examples of converting HS samples into spectrogram: (**A**) LVDD group; (**B**) control group.

The first HS (S1), systole (S), the second HS (S2), and diastole (D) are the four states of a cardiac period. Among them, the pitch of S2 is higher than that of S1, and the frequency range is 20–200 Hz [35]. To improve signal processing efficiency without sacrificing the key components of HS, firstly, the signals were resampled into 1000 Hz according to the Nyquist sampling theorem. The redundant noise was then removed using an improved wavelet denoising method [36]. Finally, the amplitude of the denoised signal $S\left(n\right)$ was normalized. The denoised waveform can be seen in the upper half of Figure 2.

(1) $$S_{norm}\left(n\right)=\frac{S\left(n\right)}{\max_{n=1}^{N}\left(\left|S\left(n\right)\right|\right)},$$

where N is the length of signal.

### 2.3. Segmentation

The logistic regression-based hidden semi-Markov model (LR-HSMM) is the perfect method for HS segmentation or identifying the beginning of cycles [37,38]. As a result, we chose the LR-HSMM to determine the boundary of S1 and S2. In a cardiac period, the mechanical activity of the heart can be captured [39]. Furthermore, the characteristics of each cardiac period may differ. Therefore, the frame length was set to 1.6 s (approximately two cardiac periods), which started with S1 onset [40]. The intervals between two frames were reserved about two periods to avoid the overlap [40]. Table 2 describes the number of HS samples in this paper.

### 2.4. Short-Time Fourier Transform

By multiplying a window function before the Fourier transform (FT), the core idea of STFT is to solve the problem that the FT could only deal with the frequency domain [31]. The window function allows STFT to represent the time and frequency domain features of the HS signal, allowing the dynamic process to be fully revealed. It is supposed that the HS signal is approximately stationary within the span of a temporal window. The 1D HS sample s(τ) is converted into the 2D spectrogram using STFT as follows:

(2) $$S(t,f)=\sum_{\tau =0}^{L-1}s(\tau )\omega (\tau -t)e^{-j2\pi \tau f},$$

where t is time, f is frequency, L is the window length, and ω(τ) is the window function. The log values of S(t,f) are represented as the 2D spectrogram.

In this paper, the Hanning window was used, and the window length was set at 256. After that, the 2D spectrogram with size of 128 × 128 was obtained, where the shades of color represent the change of signal energy of different frequency bands, as shown in Figure 2. It can be seen that the highest frequency of S2 in the LVDD group is about 150 Hz, while in the control group, it is about 200 Hz. The different features in the spectrograms have a high degree of distinction in judging whether to suffer from LVDD.

### 2.5. Convolutional Neural Network

CNN is often applied in image classification tasks, which shows better performance than traditional methods in HS signals [41,42,43]. The convolutional layer, pooling layer, and fully-connected layer are still present in CNN’s basic structure, despite the fact that it has evolved into a variety of different forms. Layer after layer, neural nodes are used to connect the layers.

The convolutional layer is to extract features. It comprises several different convolutional kernels, each of which is used to calculate different feature maps. In addition, the convolutional operation reduces the complexity of model and makes it easier to train. Equation (3) describes the jth feature map in layer l, where $x_{j}^{l}$ is calculated using the previous feature map $x_{i}^{l-1}$ multiplied by the convolutional kernel $w_{ij}^{l}$ and adding a bias parameter $b_{j}^{l}$.

(3) $$x_{j}^{l}=f\left(\sum_{i\in M_{j}}x_{i}^{l-1}\times w_{ij}^{l}+b_{j}^{l}\right),$$

where f(⋅) is activation function.

The nonlinear property gives the model the ability of uniform approximation, and the activation function can bring it into model. The expression of the ReLU is simple, as shown in Equation (4). When x≥0, the derivative of the ReLU is 1, which can solve the disappearance of gradient and the over-fitting problem.

(4) $$f\left(x\right)=\left\{\begin{array}{l} x,x\geq 0\\ 0,x<0 \end{array}\right.$$

The pooling layer, also called down-sampling, is used to reduce dimension. It moves the pooling window to output an element in the feature map according to the rules, such as max-pooling or mean-pooling. The max-pooling was used in this paper as follows:

(5) $$x_{j,k}^{l}=\max(x_{j\cdot s+m,k\cdot s+n}^{l-1}),$$

where j and k are the locations of the current feature map $x_{j}^{l}$, max is the maximum function, s stands for pooling size, m≥0, and n≤s.

The fully connected layer is essentially a perceptron connecting all the feature maps between the current layer and previous layer to generate the global semantic information. After this layer, the dropout is utilized to avoid over-fitting by disregarding some neurons at random throughout the training process [44].

In the classification task, the fully connected layer is often followed by the classification function, which is used to output the classification process of target. The commonly used classification function in CNN is softmax, which obtains the classification result by calculating the probability. The performance of softmax is improved using two fully connected layers by taking into account the influence of time and frequency in this paper [45]. The relevant information of the CNN model is shown in Table 3. The Adam optimizer with a learning rate of 0.0001 is used to improve the training speed of the model.

### 2.6. DA Methods

According to the way of generating samples, DA methods are parted into two classes: non-generative and generative methods.

#### 2.6.1. Non-Generative DA Methods

Traditional non-generative DA methods are applied to spectrogram images, such as flipping, cropping, rotation, and other operations, which will result in a large amount of information loss or distortion from spectrograms, which have no physical meaning. For audio files, five audio augmentation techniques are adopted: positive pitch shift, negative pitch shift, slow time stretches, fast time stretches, and trim silence, and then, they are converted to spectrograms, which could effectively augment audio data [46].

#### 2.6.2. Generative DA Methods

**Figure 3:**
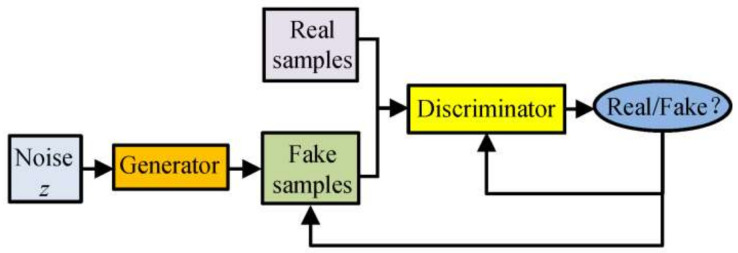
The structure of the GAN model.

Commonly used generative DA methods are the GAN model and its derivatives. The GAN model comprises two neural networks, called generator (G) and discriminator (D) [21], as shown in Figure 3.

The GAN model has the disadvantage of being too free, which results in uncontrollable and unpredictable defects when using the trained GAN model to generate samples [24]. To solve this problem, the DCGAN model has been proposed, which focus on the topology to ensure the stability in the training process [23]. In addition, since the DCGAN model can generate a large number of new samples that are closer to real samples while maintaining the validity of the semantic features [20,47], it is used to generate more samples to expand datasets in this paper. In the DCGAN model, D creates a filter that learns useful features of the target image based on CNN, while G ensures the feature quality and diversity of the generated image. The loss function of the DCGAN and GAN model is consistent, as given in Equation (6).

(6) $$\underset{G}{\min}\;\underset{D}{\max}\;V\;\left(D,G\right)\;=\;E_{x\sim P_{data}\left(x\right)}\left[\log D\left(x\right)\right]\;+\;E_{z\sim P_{z}\left(z\right)}\left[\log\left(1-D\left(G\left(z\right)\right)\right)\right],$$

where $P_{data}(x)$ represents the distribution of real samples x, and $P_{z}(z)$ represents the distribution of random noise z. G and D update parameters constantly through adversarial learning to restrict each other, and finally reach the Nash equilibrium.

In this paper, spectrograms of HS samples were used as real samples. Hence, there are 3677 images in the LVDD group and 4803 images in the control group. The DCGAN model was used to generate new samples through modeling the distribution of real samples. This is essentially a two-part minimum and maximum optimization problem:

(i) Train G: fix D and optimize the parameters of G. D(G(z)) indicates the probability of D to judge whether the sample generated by G is real or not. Hence, the larger D(G(z)), the better, implying that V(D,G) should be minimized, as shown in Equation (7). D sends its gradient back to G to update the parameters.
  (7) $$\underset{G}{\min}V\left(D,G\right)=E_{z\sim P_{z}\left(z\right)}\left[\log\left(1-D\left(G\left(z\right)\right)\right)\right]$$
(ii) Train D: fix G and optimize the parameters of D. To improve the discriminative capacity of D, the smaller the fake samples, the better, which means minimizing D(G(z)). Therefore, V(D,G) should be maximized, as shown in Equation (8). The back propagation of error updates the parameters of D.
  (8) $$\underset{D}{\max}V\left(D,G\right)=E_{x\sim P_{data}\left(x\right)}\left[\log D\left(x\right)\right]+E_{z\sim P_{z}\left(z\right)}\left[\log\left(1-D\left(G\left(z\right)\right)\right)\right]$$

A convolutional layer was added to the original DCGAN model to construct a model with an output of 128 × 128 in this paper.

(i) The generative model of the DCGAN model is a neural network similar to deconvolutional (Deconv), whose information is shown in Table 4. Its input layer is a random vector with uniform distribution. Secondly, the pooling layer is replaced by Deconv to learn spatial upsampling. Then, the stride is 2, the number of channels is cut in half, and the size is doubled. Batch normalization (BN) operation applies a transformation that maintains the means of the convolved features close to 0 and the variances of those close to 1 [48]. Adding BN operation to each layer except the output layer can solve initialization problems.
(ii) The discriminant model of the DCGAN model is CNN, whose information is shown in Table 5. Similar to the generative model, the BN operation is carried out on each layer except the input layer, which alleviates the disappearance of gradient. Leaky ReLU, an activation function based on the ReLU, assigns a non-zero slope to all negative values for preventing the sparseness of gradient [49]. A scalar with a range of 0 to 1 is output through a fully connected layer to indicate the probability that input data belong to a real sample rather than a generated sample.

**Figure 4:**
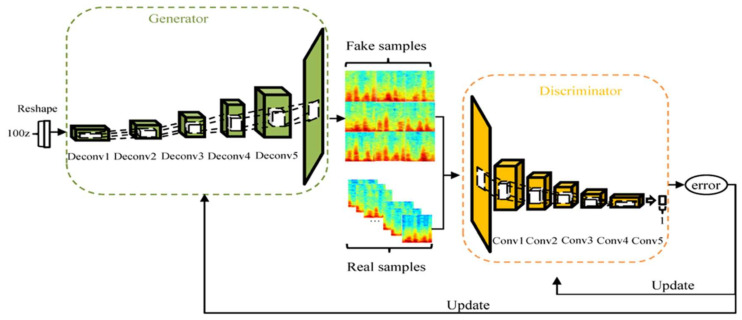
The structure of the DCGAN model.

Figure 4 depicts the structure of the DCGAN model, using Adam as the optimizer with momentum of 0.5 to help to stabilize the training; the learning rate is 0.0002, batch size is 64, epoch is 300, and the slope of Leaky ReLU is 0.2.

### 2.7. Similarity between Real and Generated Samples

For the generative result of the DCGAN model, how to evaluate whether the generated samples are qualified or not is a problem. Although the generated samples can be displayed as waveforms or spectrograms, it is difficult to detect the subtle changes visually. We presented an indirect way to measure the similarity between the samples generated by the DCGAN model and the real samples to tackle this problem [50]. To begin, the DCGAN dataset, which consists of samples generated by the DCGAN model in the LVDD and control groups, was used as a training set to train the CNN model, which learns the features that generated samples. The RS dataset, which is composed of real samples, is then utilized to test the fully trained CNN model. Finally, the test accuracy of the CNN model reflects the similarity of two types of samples. This method could be used to compare the real samples with the generated samples in order to determine the differences between the two types of samples.

## 3. Results

Matlab (Version: R2018a) software was used to implement the signal preprocessing, segmentation, and STFT. The deep learning models were built with Python (Version: 3.7.0) and the Tensorflow package (Version: 2.0.0). All experiments were implemented on a computer with an Intel Core i5-9500K processor running at 3.70 GHz and a GTX1660Ti GPU with 64 GB of RAM.

### 3.1. Result of DA

As for the results of non-generative DA methods, this paper adopted the above five audio augmentation methods to augment HS, and then converted to spectrograms through STFT. Therefore, there are 18,385 images in the LVDD group and 24,015 images in the control group, called the non-generative augmentation dataset (NG dataset), which are mixed with the RS dataset as the input of the model for LVDD diagnosis.

**Figure 5:**
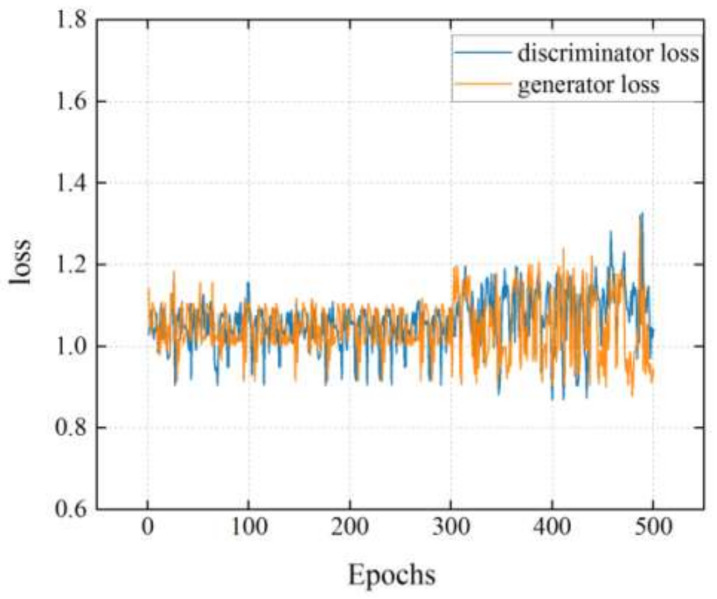
The loss value of the DCGAN model.

**Figure 6:**
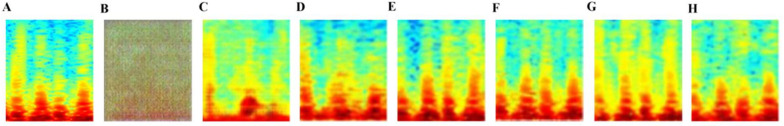
The examples of the spectrogram using the DCGAN model in the LVDD group with an increasing number of epochs: (**A**) original image; (**B**) epoch = 0; (**C**) epoch = 50; (**D**) epoch = 100; (**E**) epoch = 150; (**F**) epoch = 200; (**G**) epoch = 250; (**H**) epoch = 300.

In the DCGAN model, the cross-entropy loss function was utilized to define the loss of G and D, respectively. When the epoch was 300, the outputs of G and D were relatively stable, as well as the loss value was close to 1 and then started to oscillate, as shown in Figure 5. D can no longer tell the difference between real and fake samplesat 300th epochs. Therefore, the epoch is set to 300. Figure 6 shows the examples of the generated spectrogram using the DCGAN model in the LVDD group. First, itis an original spectrogram and then is split in intervals of 50 epochs successively, where the profile and feature of the generated spectrogram becomes clearer and more obvious with an increasing number of epochs.

### 3.2. Result of Similarity between Real and Generated Samples

**Figure 7:**
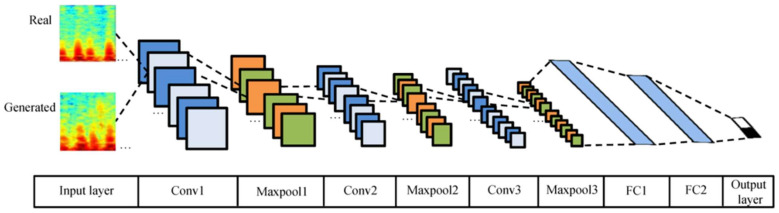
The structure of the CNN model.

The experiment was repeated by the method mentioned in Section 2.7, and the average test accuracy was 0.869. This indicates that the generated samples retain the most features of the real samples. Moreover, the DCGAN model could ensure the high validity and quality of the generated samples, regarding as a good method for DA. However, there are also some differences that indicate the diversity and unknown values of the generated samples. In view of this, it is also possible to mix the DCGAN dataset with the RS dataset and use them as the input of the CNN model (or other models) to identify LVDD. The structure of the CNN model is depicted in Figure 7.

### 3.3. Identification Performance

The performance of the proposed method was calculated from the testing set. All the samples were split into ten equal-sized subsets. Eight subsets were selected as the training set, and one subset was used as the validation set to tune the parameters of training process, and the rest was selected as the testing set, while specifying that the samples from the same subject cannot appear in the different subsets of the 10-fold cross-validation scheme. This process was carried out ten times. The model was evaluated in this paper using the typical evaluation metrics of accuracy (Acc), sensitivity (Se), and specificity (Sp).

(9) $$Acc=\frac{TP+TN}{TP+TN+FP+FN};$$

(10) $$Se=\frac{TP}{TP+FN};$$

(11) $$Sp=\frac{TN}{TN+FP},$$

where TP = true positive, TN = true negative, FP = false positive, and FN = false negative.

#### 3.3.1. Expansion Coefficient in the DCGAN Dataset

**Figure 8:**
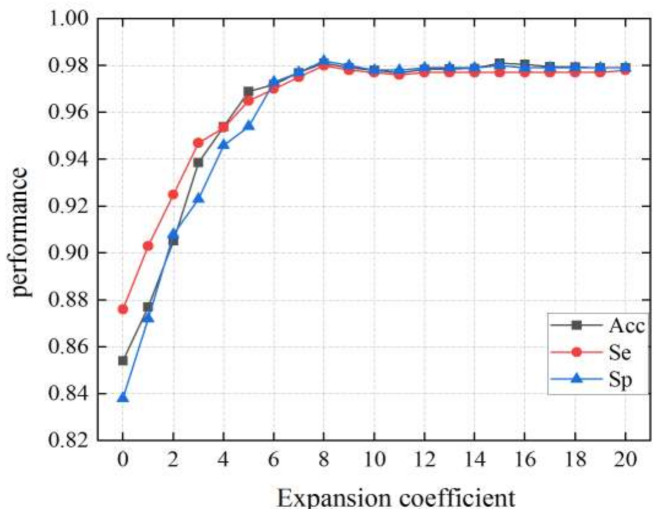
The performance of the CNN model in the DCGAN dataset with different coefficients.

The expansion coefficient represents the average number of images generated by DA from each spectrogram of the RS dataset. According to Figure 8, when the expansion coefficient ranges 0 to 8 times, the performance is significantly improved, and the over-fitting problem is gradually solved with the expansion of samples and the use of dropout operation. When the expansion coefficient in the DCGAN dataset is set to 8 times, the CNN model achieves good performance with an Acc of 0.981, a Se of 0.980, and a Sp of 0.982. As samples are further expanded, when the coefficient is in the range 8 to 20 times, the performance does not increase, reaching a state of saturation. Therefore, there are 29,416 images in the LVDD group and 38,424 images in the control group in the DCGAN dataset.

#### 3.3.2. Performance Comparison

In the three different datasets, we tested the performance of the CNN model. Moreover, the CNN model was compared against VGG-16, VGG-19, ResNet-18, ResNet-50, DenseNet-121, and AlexNet to determine the best model for the diagnosis of LVDD. The number of parameters of these models is shown in Table 6. The global average pooling layer was adjusted before the fully connected layer to avoid dimension mismatches, and the hyperparameters of each model remain consistent.

The CNN model was trained for 500 epochs, and the gradient of backpropagation was also calculated using the cross-entropy loss function. After expanding the samples, the dropout operation in the CNN model achieved a certain effect of regularization, accelerating the convergence speed.

According to Table 7, Table 8 and Table 9, our proposed CNN model achieves the best classification performance in the RS dataset + DCGAN dataset, with an average Acc of 0.987, Se of 0.986, and Sp of 0.988 among the seven models. The CNN model has the fewest parameters, so it takes less time to train. It can be seen that the length of training time has little impact on model performance. Furthermore, the DCGAN-based generative DA method outperforms the non-generative DA method in terms of classification performance. Compared with the RS dataset, the Acc, Se, and Sp of the CNN model in the RS dataset + DCGAN dataset are improved by 0.071, 0.054, and 0.093, respectively, which shows that the DCGAN model could effectively expand HS dataset of LVDD, thus improving the performance for LVDD diagnosis.

**Figure 9:**
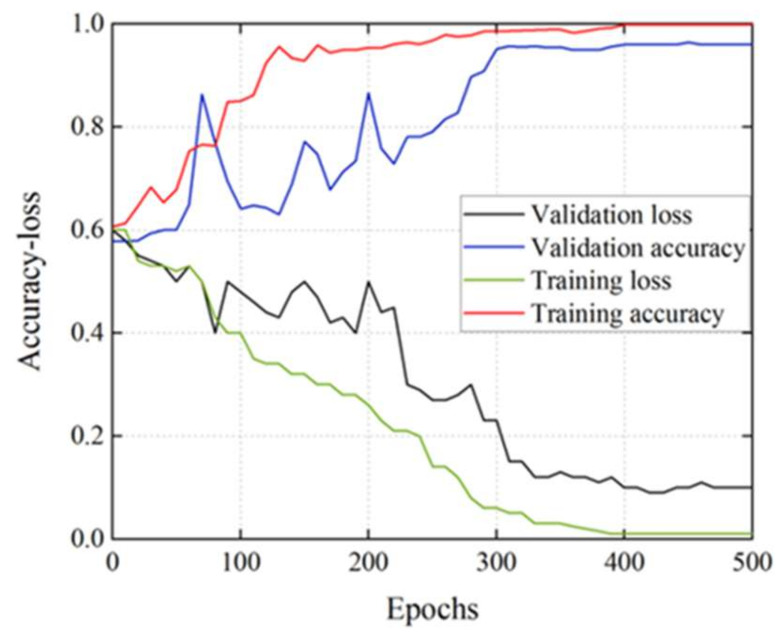
The training and validation performance of the CNN model in the RS dataset + DCGAN dataset at 500 epochs.

Figure 9 demonstrates how the performance of the CNN model in the RS dataset + DCGAN dataset improves as the number of epochs increases during training. Since the 350th epoch, the CNN model has steadily converged, with the accuracy and loss of validation set approaching those of the training set, indicating that the training epoch of 500 is sufficient.

## 4. Discussion

Over the years, many studies on the diagnosis of LVDD involved biomarkers, echocardiographic diagnostic indices, epidemiology, and pathogenesis. For example, Mocan et al. [51] found that IL-6 as an inflammatory biomarker has the best predictive capacity for LVDD, but such an invasive method is not suitable for pervasive application. HS signal classification, a non-invasive method, has potential to identify of cardiovascular diseases and has been extensively studied, but the objects of these researches are different [52,53]. However, the study for LVDD diagnosis based on deep learning using HS has not been studied. This paper could therefore be an efficient supplement for the diagnosis of LVDD.

Due to the available HS databases are insufficient to train deep learning models, Narváez and Percybrooks [22] developed a GAN-based DA method that could only generate normal HS with a single type and participate in classification tasks. In the GAN model, G and D are in healthy competition for concurrent learning. The loss function is derived using the output of D, and t hereforeit updates parameters more quickly, whereas G does not always converge. Another drawback of the GAN model is that, for diverse random inputs, G produces similar outputs [54]. In the original DCGAN model, CNN architecture is integrated into unsupervised learning training to alleviate this problem and boost generative effect [55]. In this paper, the DCGAN model was applied to the analysis of HS, which could solve the problem of sparsity and imbalance of samples using the DA of HS dataset. A DCGAN model with the output of 128 × 128 was constructed to automatically generate spectrograms of HS frames, which could be used as the input of the different deep learning models for training. Compared with the non-generative DA method, the DCGAN-based DA method improves the classification performance of different models. Moreover, it is also beneficial to enhance the speed and quality of the generated samples, which shows the feasibility and effectiveness of the DCGAN model for DA in the HS field.

The benefits of CNN can be shown in the following areas: it could learn features from datasets automatically, and it has convolutional invariant, and it could obtain features straight from the convolutional layer, overcoming the limitations of hand-crafted feature extraction and providing high reliability [56]. Furthermore, transforming the HS signal into a 2D spectrogram could better describe the time and frequency aspects of the signal [57]. Based on the above advantages, we proposed a 2D CNN model for the diagnosis of LVDD. The performance of this model is compared with six common deep learning models, as shown in Table 7, Table 8 and Table 9. In the RS dataset + DCGAN dataset, the CNN model has the best classification performance and stability, which is a wonderful solution to the problem of small samples. The method classifies spectrograms generated using the DCGAN model and decreases the influence of out-of-distribution inputs, indicating it the preferred method for the diagnosis of LVDD using HS.

This paper also has two limitations. Firstly, the samples generated by the DCGAN model have a low resolution of 128 × 128, which may affect the classification results of deep learning models [58]. Secondly, the number of collected HS signals of LVDD is small, which makes the features in the spectrograms insufficient to continue DA effectively to improve model performance. We could collect more HS signals of LVDD to train the super resolution generative adversarial networks (SRGAN) model to generate high-resolution spectrograms, so as to have a better performance of deep learning models.

The DA method proposed in this paper could be utilized in the future to establish HS databases for the other cardiovascular diseases in order to expand the application of deep learning in the different fields. It provides important auxiliary diagnosis for medical professionals and forms a non-invasive, low-cost, and reusable early diagnosis method to avoid tedious examination.

## 5. Conclusions

Early diagnosis of LVDD can help guide treatment and provide important insights into the progression and severity of HFpEF. We proposed a CNN model for the diagnosis of LVDD using HS in this paper that does not rely on hand-crafted feature extraction, as well as a DCGAN-based DA method for expanding the HS dataset of LVDD. The experimental results support the efficacy of our proposed model. Furthermore, the suggested method is not constrained by small datasets and offers a viable solution for HS signal analysis using deep learning, which is a potential non-invasive technology for the identification of LVDD.

## Figures and Tables

**Table 1:** Some Doppler echocardiography indices (mean ± standard deviation).

Index	LVDD Group	Control Group	*p*-Value
LVEF	0.45 ± 0.16	0.64 ± 0.03	0.000 *
Peak E-wave velocity(cm/s)	73.81 ± 26.60	63.25 ± 14.07	0.034 *
Peak A-wave velocity(cm/s)	72.58 ± 27.48	75.53 ± 18.07	0.589
Septal e’ velocity(cm/s)	4.18 ± 1.40	6.20 ± 2.09	0.000 *
Average E/e’	18.57 ± 6.55	10.66 ± 2.00	0.000 *
LA volume index (mL/m^2^)	40.68 ± 15.49	23.52 ± 5.48	0.000 *
TR velocity(m/s)	28.74 ± 6.62	16.79 ± 7.26	0.000 *

* *p*-value < 0.05 demonstrates statistically significant difference.

**Table 2:** The number of HS samples in different groups.

Group	Subjects	Age (Mean ± Square Deviation)	Samples	Sample Length (s)
LVDD group	30	Aged 24–89 (66.87 ± 16.21)	3677	1.6
Control group	41	Aged 19–81 (58.71 ± 13.19)	4803	1.6

**Table 3:** The detailed information of the CNN model.

#	Layer	Filter Size	Stride	Output Dimension	Activation Function
1	Input	—	—	(128, 128, 3)	—
2	Conv1	3 × 3	1	(128, 128, 64)	ReLU
3	Maxpool1	2 × 2	2	(64, 64, 64)	—
4	Conv2	3 × 3	1	(64, 64, 32)	ReLU
5	Maxpool2	2 × 2	2	(32, 32, 32)	—
6	Conv3	3 × 3	1	(32, 32, 16)	ReLU
7	Maxpool3	2 × 2	2	(16, 16, 16)	—
8	FC1	—	—	(128, 1)	—
9	FC2	—	—	(100, 1)	dropout = 0.5
10	Output	—	—	(0, 1)	softmax

Conv = convolutional layer; Maxpool = max-pooling layer; FC = fully connected layer.

**Table 4:** The detailed information of the generative model.

Layer	Filter Size	Stride	Output Dimension	Activation Function	BN
Input	—	—	(1, 1, 100)	ReLU	Yes
Deconv1	5 × 5	2	(8, 8, 512)	ReLU	Yes
Deconv2	5 × 5	2	(16, 16, 256)	ReLU	Yes
Deconv3	5 × 5	2	(32, 32, 128)	ReLU	Yes
Deconv4	5 × 5	2	(64, 64, 64)	ReLU	Yes
Deconv5	5 × 5	2	(128, 128, 3)	ReLU	Yes
Output	—	—	(128, 128, 3)	Tanh	No

Deconv = deconvolutional layer.

**Table 5:** The detailed information of the discriminant model.

Layer	Filter Size	Stride	Output Dimension	Activation Function	BN
Input	—	—	(128, 128, 3)	Leaky ReLU	No
Conv1	5 × 5	2	(64, 64, 64)	Leaky ReLU	Yes
Conv2	5 × 5	2	(32, 32, 128)	Leaky ReLU	Yes
Conv3	5 × 5	2	(16, 16, 256)	Leaky ReLU	Yes
Conv4	5 × 5	2	(8, 8, 512)	Leaky ReLU	Yes
Conv5	5 × 5	2	(4, 4, 1024)	Leaky ReLU	Yes
Output	—	—	(0, 1)	sigmoid	Yes

Conv = convolutional layer.

**Table 6:** Characteristics of the CNNs’ architectures used in this paper.

Models	Parameters	Epochs
VGG-16	138,357,544	500
VGG-19	20,483,904	500
ResNet-18	63,470,656	500
ResNet-50	46,159,168	500
DenseNet-121	62,378,344	500
AlexNet	82,378,344	500
Proposed CNN	559,396	500

**Table 7:** The results of different models on the testing set in the RS dataset during the 10-fold cross-validation (mean ± standard deviation).

Models	Evaluation Metrics			Training Time (mins:secs)
	Acc	Se	Sp	
VGG-16	0.913 ± 0.018	0.929 ± 0.018	0.892 ± 0.019	443:68
VGG-19	0.894 ± 0.022	0.908 ± 0.021	0.876 ± 0.022	112:37
ResNet-18	0.861 ± 0.021	0.883 ± 0.020	0.864 ± 0.020	251:26
ResNet-50	0.883 ± 0.022	0.899 ± 0.021	0.871 ± 0.020	178:13
DenseNet-121	0.842 ± 0.019	0.856 ± 0.022	0.825 ± 0.021	224:79
AlexNet	0.879 ± 0.017	0.897 ± 0.019	0.857 ± 0.019	317:25
Proposed CNN	0.916 ± 0.015	0.932 ± 0.017	0.895 ± 0.018	65:44

RS dataset = 3677 images in the LVDD group and 4803 images in the control group.

**Table 8:** The results of different models on the testing set in the RS dataset + NG dataset during the 10-fold cross-validation (mean ± standard deviation).

Models	Evaluation Metrics			Training Time (mins:secs)
	Acc	Se	Sp	
VGG-16	0.949 ± 0.009	0.958 ± 0.009	0.931 ± 0.010	535:25
VGG-19	0.928 ± 0.013	0.933 ± 0.012	±0.912 ± 0.013	243:14
ResNet-18	0.902 ± 0.011	0.911 ± 0.010	0.901 ± 0.011	361:12
ResNet-50	0.925 ± 0.012	0.934 ± 0.011	0.914 ± 0.011	302:57
DenseNet-121	0.887 ± 0.011	0.896 ± 0.012	0.867 ± 0.012	332:13
AlexNet	0.919 ± 0.009	0.930 ± 0.009	0.898 ± 0.010	435:79
Proposed CNN	0.955 ± 0.005	0.966 ±0.007	0.947 ± 0.008	179:26

RS dataset + NG dataset = 22,062 images in the LVDD group and 28,818 images in the control group.

**Table 9:** The results of different models on the testing set in the RS dataset + DCGAN dataset during the 10-fold cross-validation (mean ± standard deviation).

Models	Evaluation Metrics			Training Time (mins:secs)
	Acc	Se	Sp	
VGG-16	0.981 ± 0.003	0.978 ± 0.004	0.979 ± 0.003	494:56
VGG-19	0.964 ± 0.004	0.956 ± 0.005	0.961 ± 0.005	194:78
ResNet-18	0.957 ± 0.004	0.941 ± 0.006	0.949 ± 0.005	314:34
ResNet-50	0.968 ± 0.005	0.952 ± 0.006	0.951 ± 0.006	263:71
DenseNet-121	0.936 ± 0.005	0.922 ± 0.007	0.913 ± 0.006	286:45
AlexNet	0.962 ± 0.004	0.959 ± 0.005	0.955 ± 0.005	381:24
Proposed CNN	0.987 ± 0.001	0.986 ± 0.002	0.988 ± 0.002	130:13

RS dataset + DCGAN dataset = 33,093 images in the LVDD group and 43,227 images in the control group.

## Data Availability

The data presented in this study are available on request from the corresponding author. The data are not publicly available due to privacy and ethical restrictions.
